# Quantitative margin assessment of radiofrequency ablation of a solitary colorectal hepatic metastasis using MIRADA RTx on CT scans: a feasibility study

**DOI:** 10.1186/s12880-019-0360-2

**Published:** 2019-08-20

**Authors:** B. G. Sibinga Mulder, P. Hendriks, T. R. Baetens, A. R. van Erkel, C. S. P. van Rijswijk, R. W. van der Meer, C. J. H. van de Velde, A. L. Vahrmeijer, J. S. D. Mieog, M. C. Burgmans

**Affiliations:** 1 Department of Surgery, Leiden University Medical Center, Leiden, The Netherlands; 2 Department of Radiology, Leiden University Medical Center, Leiden, The Netherlands

**Keywords:** Radiofrequency ablation, Colorectal liver metastases, Local recurrence, Co-registration

## Abstract

**Background:**

Compared to surgery, radiofrequency ablation(RFA) for colorectal liver metastasis(CRLM) is associated with higher local recurrence(LR) rates. A wide margin (at least 5 mm) is generally recommended to prevent LR, but the optimal method to assess ablation margins is yet to be established. The aim of our study was to evaluate the feasibility and reproducibility of CT-CT co-registration, using MIRADA software, in order to assess ablation margins of patients with CRLM.

**Methods:**

In this retrospective study, pre- and post-ablation contrast-enhanced CT scans of 29 patients, treated with percutaneous RFA for a solitary CRLM, were co-registered. Co-registration was performed by two independent radiologist, based on venous structures in proximity to the tumor. Feasibility of CT-CT co-registration and inter-observer agreement for reproducibility and ablation margins was determined. Furthermore, the minimal ablation margin was compared with the occurrence of LR during follow-up.

**Results:**

Co-registration was considered feasible in 18 patients (61% male, 63.1(±10.9) year), with a perfect inter-observer agreement for completeness of ablation: κ = 1.0(*p* < 0.001). And substantial inter-observer agreement for measurement of the minimal margin (≤ 0 mm, 1-5 mm, ≥ 5 mm): κ = 0.723(*p*-value < 0.001). LR occurred in eight of nine(88.9%) incompletely ablated CRLM and in one of the nine completely ablated CRLM(11.1%).

**Conclusion:**

Co-registration using MIRADA is reproducible and potentially a valuable tool in defining technical success. Feasibility of co-registration of pre- and post-ablation CT scans is suboptimal if scans are not acquired concordantly. Co-registration may potentially aid in the prediction of LR after percutaneous ablation.

**Electronic supplementary material:**

The online version of this article (10.1186/s12880-019-0360-2) contains supplementary material, which is available to authorized users.

## Background

Radiofrequency ablation is an established minimally invasive treatment for patients with unresectable liver metastases from colorectal cancer [1]. Compared to surgery, RFA is associated with higher rates of local recurrence (LR) [2, 3]. Histological confirmation of treatment success is not possible, therefore, technical success of ablation is generally defined as having successfully delivered a certain amount of energy that is considered to create an ablation zone with sufficient margins [4]. In clinical practice, CT is most commonly used for margin evaluation based on visual qualitative assessment without quantitative determination of the ablation margin. Generally, a margin of at least 5 mm is advised [5]. This seems intuitively correct, but there is only limited evidence to support this assumption and no standardized and validated method to assess ablation margins [6, 7].

Co-registration of pre- and post-ablation cross-sectional images allows three-dimensional quantitative assessment of ablation margins. Several studies investigating different techniques for quantitative assessment of RFA procedures for hepatocellular carcinoma (HCC) using CT-CT co-registration have been performed and are found to be superior to qualitative visual assessment in determining ablation margins and predicting local tumor recurrence [7–9]. Although, different results are obtained between experienced readers and less experienced readers. Quantitative assessment offers a more objective and reproducible method to evaluate technical success of ablation [8].

Co-registration of pre- and post-ablation CT scans is challenging, as shape and position of the liver may differ between the two scans as a result of differences in patient positioning, breathing related liver motion and liver deformation due to the ablation or previous surgery [10]. Most common co-registration algorithms are rigid, and/or need manual manipulation. By using a semi-automatic, non-rigid registration algorithm, an automatic correction for difference in liver position and morphology can be applied.

In this retrospective study, we evaluated feasibility and reproducibility of quantitative ablation margin assessment of patients with a solitary CRLM, using the described CT-CT co-registration software from Mirada RTx. Furthermore, we correlated the minimal ablation margins to local tumor recurrence.

## Methods

The study was designed as a retrospective cohort study to investigate the feasibility and reproducibility of co-registration of pre- and post-ablation CT-scans of patients with a solitary CLRM. Two independent readers assessed the co-registration and a scoring model was used to determine feasibility and reproducibility. Furthermore, the minimal margins obtained after ablation were measured and correlated to local recurrence.

### Medical ethical approval

For this study medical ethical approval was obtained. All patient gave informed consent to undergo RFA. Informed consent was waived for the conduct of the study. Patient confidentiality was guaranteed using anonymized data and radiologic images, and all data was entered into an encrypted and secured database.

### Patients

Between January 2009 and March 2014, 313 patients underwent a first thermal ablation procedure of the liver in our institution (re-ablations were not included). Of the 313 patients, 284 patients were excluded from the analyses for the following reasons: hepatocellular carcinoma (*n* = 112), liver metastases other than CRLM (*n* = 25), a pre-ablation MRI scan (*n* = 100), microwave ablation (*n* = 8), ablation during open surgery (*n* = 19), multifocality (*n* = 13), technical failure (*n* = 3) or a missing post-ablation CT (*n* = 4). Co-registration of liver images of two scans is often challenging due to alterations in liver shape and position: accurate co-registration of a liver area often results in a mismatch in other areas. In order not to increase the complexity of co-registration, patients with more than 1 lesion were not included in this feasibility study. Finally, we included 29 patients who underwent percutaneous radiofrequency ablation (RFA) of a solitary CRLM. Patient and lesion characteristics are shown in Additional file 1: Table S1. Pre- and post-ablation contrast-enhanced CT scan, including at least an portal-venous phase, was available in all patients with the baseline CT scan being performed within 2 months prior to the procedure.

### RFA procedure

Three interventional radiologists specialized in RFA of the liver performed the RFA procedures and had at start of the inclusion period an experience of at least 2 years. Percutaneous RFA was performed under general anesthesia under ultrasound guidance and in case of suboptimal ultrasonic guidance the procedure was performed with CT guidance. A single electrode was used (3 cm exposed tip Cooltip (Covidien, Gosport Hamspire, United Kingdom) or StarBurst XL (AngioDynamics, Amsterdam, Netherlands)) or multiple electrodes with a switch-control system (3 or 4 cm exposed tip Cooltip). Ablation was performed for 12 (single Cooltip electrode) or 16 min (multiple Cooltip electrodes) using standard impedance controlled ablation. Temperature-based ablation was performed with the StarBurst XL electrode.

Contrast-enhanced CT was performed immediately after ablation on a 16-slice spiral CT (Aquillion-16, Toshiba, Tokyo, Japan) using the following scanning parameters: 16 × 1 mm scanning, 120 KV, rotation 0.5 s, contrast Ultravist 370 (dose weight depended) with a delay of 75 s for portal venous phase. In the Additional file 1: Table S2 the scanning protocol is included.

All RFA procedures were deemed to be technically successful at the time of the procedure if 1) a predefined amount of energy (based on information provided by vendors on the ablation size at different settings) was successfully delivered to the tumor and 2) the coagulation area was thought to fully encompass the tumor based on visual qualitative assessment and 3) residual tumor enhancement on immediate post-ablation CT was absent. Visual assessment was performed by eye-balling and two-dimensional measurements. Pre- and post-ablation CT scans were projected side-by-side on the computer screen. By scrolling up and down both scans the interventional radiologist assessed whether the ablation area was correctly located and was thought to fully encompass the tumor with a margin of at least 5 mm. Also, the post-ablation scan was assessed to rule out residual tumor enhancement. In addition to this, the distance was measured of the tumor and ablation zone to anatomical landmarks such as the liver edges and veins in order to confirm that the ablation zone was in a correct position and ablation margins were considered to be sufficient.

### Mirada RTx software

Mirada RTx is a software application developed for radiation therapy treatment planning. The software is integrated into Vitrea Advanced Visualisation (Vital Images, Minnetonka, U.S.A) and designed for rigid and deformable registration of medical image datasets including PET, CT, MR, and SPECT. In our study, we used Mirada RTx to perform CT-CT co-registration with a semi-automated deformable registration algorithm with manual alteration when necessary. These manual alterations were either done by rotation and translation of a scan, or with use of a landmark algorithm that interpolates a deformable transformation by manually selecting corresponding anatomical landmarks in both scans. Delineation of the tumor volume and ablation area was done using a greyscale-based semi-automatic delineation tool with manual adjustments for accurate segmentation. In a fused-imaging view, RFA margins were quantitatively assessed by expanding the tumor’s contour until line intersection with the delineated ablation area. In case the tumor was not located completely within the ablation area, negative margin size was determined in the same way by expanding the ablation area delineation. Besides the narrowest margin in millimeters (mm), the anatomical location of the narrowest margin was determined as well. In case the tumor exceeded the tumor ablation area, the anatomical location of the highest tumor excess was recorded.

### Scoring

Two interventional radiologists of the LUMC staff, experienced in RFA of liver lesions, who were blinded to follow-up data, independently performed co-registration of the pre- and postablation CT scans. Pre- and post-ablation portal venous phase CT scans were loaded into Mirada RTx. First, manual co-registration of the pre- and post-ablation CE-CT scans (venous phase) was based on venous structures and other liver landmarks in proximity to the tumor, such as cysts or calcifications. Landmarks were placed on bi- or trifurcation of the portal vein to co-register the pre- and post-ablation CT. At the start of the co-registration process landmarks were placed centrally. Then, peripheral landmarks were chosen that were located closer to the tumor. After co-registration was performed a grading system from 1 to 5 was used to assess the reliability of co-registration: 1 = co-registration completely unreliable due to large differences in liver shape and position between pre- and post-ablation CT, 2 = suboptimal co-registration, 3 = sufficient co-registration, but not accurate for measurement in mm, 4 = good co-registration or 5 = perfect co-registration. If the quality of co-registration in a patient was graded 4 or 5 by both radiologists, tumor and ablation volumes were delineated on both scans using automatic contour detection. In case the observers graded co-registration of the patient different, a consensus reading was applied.

Secondly, the automatic contour detection was evaluated by the radiologist and manually altered in case contour detection was not considered to be inaccurate. Both delineations (tumor and ablation zone) were projected in one scan, resulting in an overlay of pre- and post-ablation CT scans. This overlay allowed assessment of ablation margins. The side of the minimal margin was noted, and the margin was measured in millimeters by both observers. In case of incomplete ablation, the extension of the tumor beyond the ablation zone was recorded in millimeters (negative margin). Inter-observer agreement was calculated for two different outcomes: 1) total encompassment of the tumor by the ablation zone; meaning that the tumor was inside the ablation zone, or without total encompassment of the tumor; meaning that the ablation zone did not cover the tumor completely, and 2) negative or no margin (≤0 mm), a minimal margin of 1–5 mm or a minimal margin of ≥5 mm. In case the observers measured different margins for the same patient, resulting in a difference in category grouping, a consensus reading was applied.

### Local recurrence

Follow-up was performed according to standard local protocol, including a visit every 3 months to the surgical outpatient clinic, carcinoembryonic antigen (CEA) determination, and a CE-CT scan of the chest and abdomen. The follow-up scans were evaluated by an independent radiologist unaware of the ablation margin that was obtained during the ablation. In case of reported recurrence, the side of insufficient or minimal margin was compared with the localization of LR during follow-up. Patient and lesion characteristics of the patients without LR were compared with the patients with LR. The average of the minimal obtained margin determined by both observers was compared to development of LR.

### Statistics

The level of agreement in margins between the two observers was estimated using unweighted Kappa κ statistics. The agreement was interpreted as poor (0), slight (≤ 0 to 0.20), fair (0.21 to 0.40), moderate (0.41 to 0.60), substantial (0.61 to 0.80), almost perfect (≥ 0.80 to 0.99) and perfect (1.00) [11]. For continuous data, groups were compared using the independent t-test; categorical data were compared using the chi-square test. Data were analyzed using SPSS version 23.0. The statistical results were considered to indicate significance if the *P*-value was less than 0.05.

## Results

All tumor ablations (*n* = 29) (median size 21 mm (range 8-42 mm)) were considered to be technically successful at the time of the procedure, as judged qualitatively by the radiologist performing the procedure. Twenty tumors were ablated ultrasound guided, in nine patients the tumors could not be visualized with ultrasound, their ablation was performed CT guided.

### Scoring

The co-registration of pre- and post-ablation CT scans was good (4) or perfect (5) in 18 (62%) patients. These patients were included in further analyses and for correlation with local recurrence. Patient and lesion characteristics of the patients are presented in Table 1. No significant differences in patient and lesion characteristics were observed between the patients who developed LR and who did not develop LR (Table 1).

**Table 1:** Patient characteristics

		total		no LR		LR		*p-value*
		*n*		*n*		*n*		
total		18		9		9		
age	mean (SD)	63.1	±10.9	61.8	±7.6	64.3	±13.8	*0.64*
sex	male	11	61%	6	67%	5	56%	*0.63*
	female	7	39%	3	33%	4	44%	
previous CRLM surgery	yes	9	50%	5	56%	4	44%	*0.64*
	no	9	50%	4	44%	5	56%	
occurrence of CRLM	metachronous	7	39%	3	33%	4	44%	*0.63*
	synchronous	11	61%	6	67%	5	56%	
days preoperative CT scan - RFA procedure	mean (SD)	32.2	±17.2	27.9	±15.8	36.4	±18.4	*0.31*
year of RFA	2009–2011	7	39%	4	44%	3	33%	*0.63*
	2012–2014	11	61%	5	56%	6	67%	
lesion size (mm)	median (range)	22	8–42	22	8–31	25	11–42	*0.17*
categorized lesion size	< 21 mm	8	44%	4	44%	4	44%	*1.00*
	> 20 mm	10	56%	5	56%	5	56%	
liver half and segment								
left	segment 2	1	6%			1	11%	*0.14*
	segment 3	1	6%	1	11%			
	segment 4	5	27%	1	11%	4	45%	
right	segment 5	2	11%	1	11%	1	11%	
	segment 6	1	6%			1	11%	
	segment 7	4	22%	3	33%	1	11%	
	segment 8	4	22%	3	33%	1	11%	
follow-up (months)	mean (SD)	44.7	±20.5	52.3	±14.8	37.3	±23.5	*0.13*
survival	death	5	28%	1	11%	4	44%	*0.11*

*CRLM* colorectal liver metastasis, *RFA* radiofrequency ablation

The cause for suboptimal co-registration (grade 1, 2 or 3) was the difference in liver position during the pre- and post-ablation scan. This could be due to a difference in position of the patient (diagnostics scans were acquired with the patient in a supine position, whereas some RFA procedures were performed with the patient in a left lateral position to allow a lateral intercostal approach) or because the scans were obtained during a different breathing phase (in- or expiration). In two of the excluded patients there were artefacts in the post-ablation scans, therefore co-registration was not possible (grade 1).

Eventually, 10 patients were graded 4 and 8 patients were graded 5. In 8 patients consensus reading was performed.

Based on quantitative analysis using CT-CT fusion imaging, coverage of the tumor by the coagulation area was found to be incomplete in tumors of 9 patients (50%). In the other 9 patients complete ablation was achieved with a mean ablation margin of 2.2 ± 1.9 mm.

The inter observer agreement was perfect for completeness of the ablations of the 18 CRLM patients: κ = 1.0 (*p*-value < 0.001). Agreement for measurement of the minimal margin (mm), divided in three groups (≤ 0 mm, 1-5 mm, ≥ 5 mm), was substantial: κ = 0.723 (p-value < 0.001). Eventually, 13 patients were in the ≤0 mm group, six in the 1-5 mm group, and two in the ≥5 mm group. In 3 patients a consensus reading was performed.

### Local recurrence rate

**Fig. 1:**
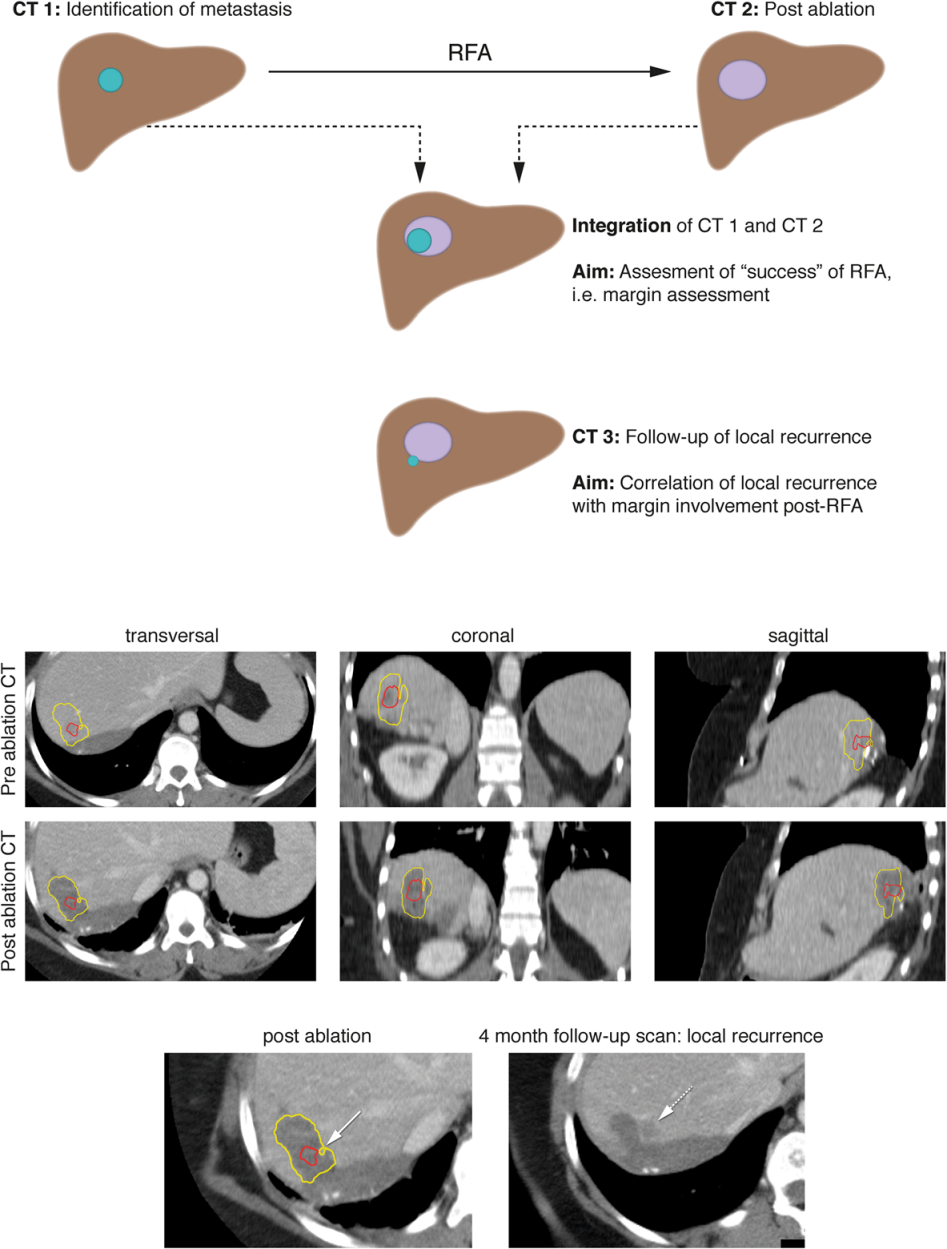
Schematic overview and real-life implementation of the used technique for assessment of successful ablation. Panel above: schematic overview of technique. Panel below: example of a patient who developed local recurrence (dashed arrow) of her colorectal liver metastasis 4 months after ablation, precisely on the spot of the minimal obtained margin (arrow). The patient has undergone previous liver surgery, the clips are used as landmark

**Fig. 2:**
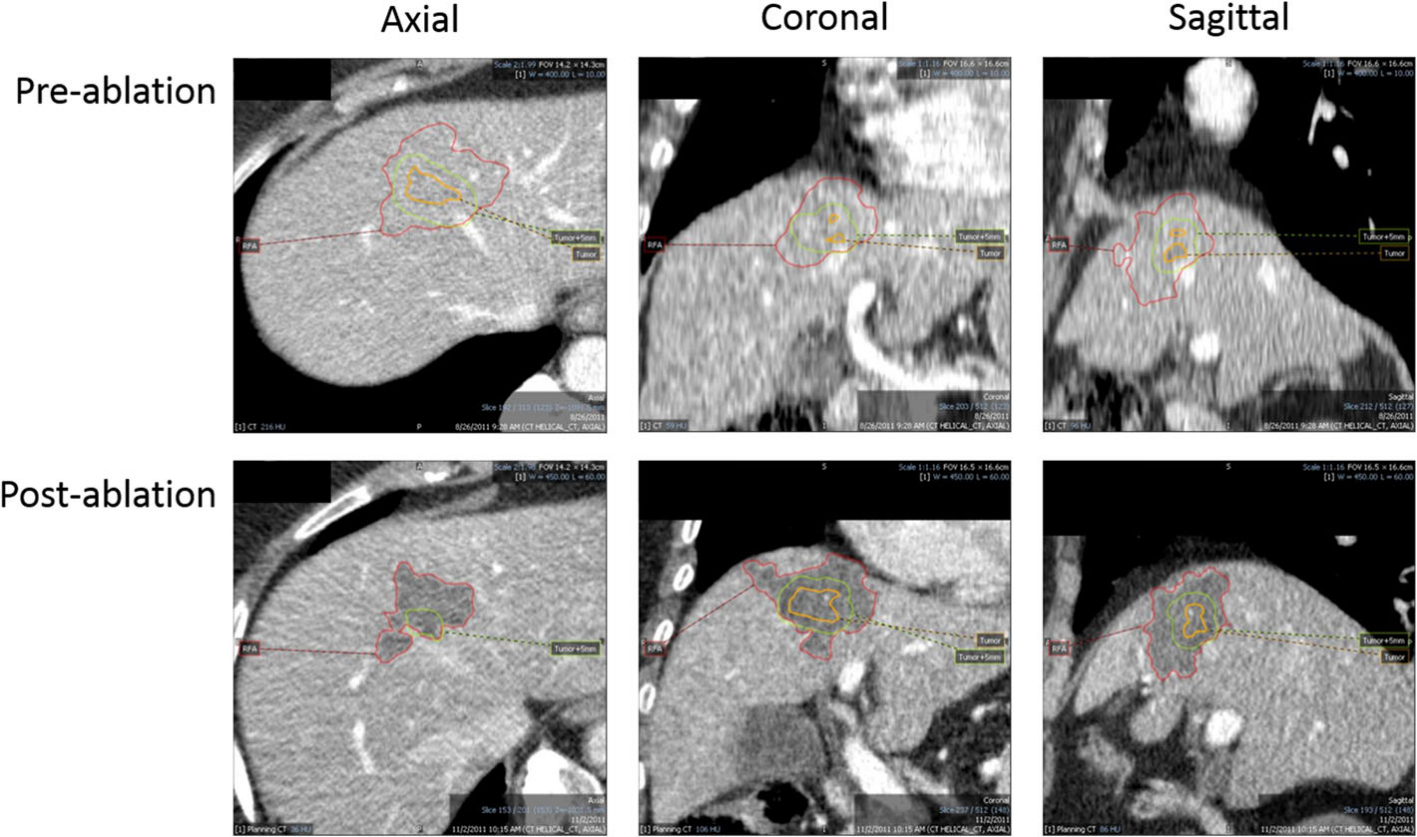
Overview in all dimensions after co-registration and delineation of the tumor and ablated zones. Orange delineation: tumor; green delineation: tumor + 5 mm; red delineation: ablation zone

A schematic overview of the technique and the real-life implementation, including an example of a patient with LR 4 months after ablation is given in Fig. 1. In Fig. 2 an overview is demonstrated of the delineation of a tumor and ablation zone after co-registration was performed.

Patients with incomplete tumor ablation based on quantitative assessment were at a significantly higher risk of LR: 88.9% compared to 11.1% patients in whom complete ablation was successfully achieved (*p*-value = 0.003). LR occurred after a median of 7 months (range 3–9).

**Fig. 3:**
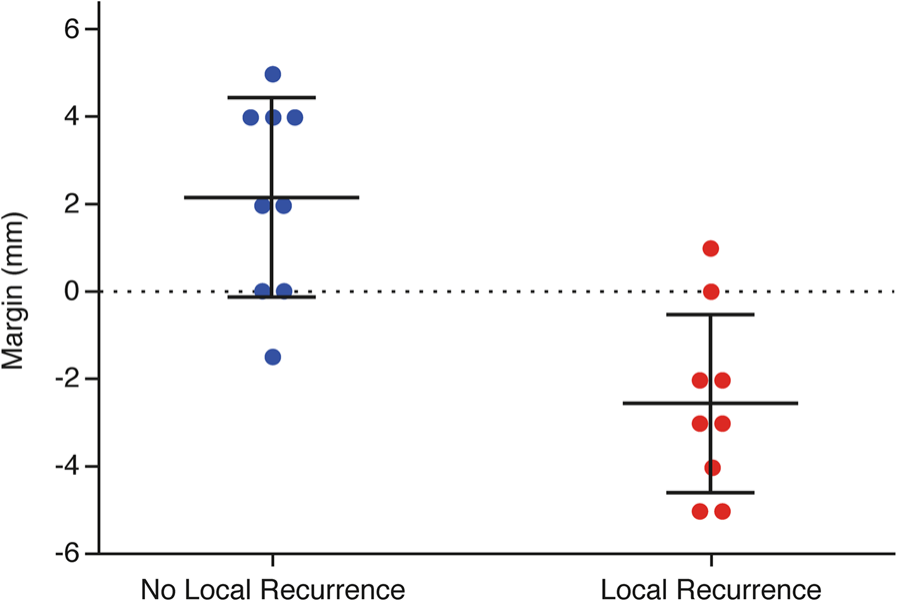
The obtained average minimal margin (in mm) correlated to the presence of local recurrence

The correlation between the minimal obtained margin and the presence of LR was also determined: the average obtained margin of patients without LR was 2.1 mm compared to an average obtained margin of − 2.6 mm for patients with LR (*p*-value < 0.001). The minimal obtained margins (average of the measured margins by the observers) correlated to the presence of LR is demonstrated in Fig. 3.

## Discussion

In our study the obtained percutaneous RFA margins in patients with a solitary CRLM were analyzed using CT-CT co-registration software with Mirada RTx. The main objective of this study was to evaluate the feasibility and reproducibility of this technique. From the 29 patients that were included, sufficient co-registration was only possible in 18 patients (62%). The reason for suboptimal co-registration in the remaining 11 patients is due to difference in liver position and/or shape. This can be explained by the retrospective study design, in which patient positioning and breathing related liver motion were not taken into account during CT acquirement. Despite the limited feasibility, our results demonstrate that quantitative analyses of ablation margins with Mirada software may be a valuable method to determine the end-point of ablation. In those patients in whom co-registration was feasible, the inter-observer agreement for completeness of the ablation of the patients whose scan were co-registered adequately, was perfect. In addition, a correlation was found between completeness of ablation and local recurrence. The good reproducibility of this technique and trend to the possibility of predicting local recurrence is an important finding for future prospective studies.

Overall, this quantitative method of margin measurement was accurate for patients who had scans with concordant liver positions. Against our expectations, half of the evaluated patients were unsuccessfully ablated when re-assessed using quantitative co-registration software. By using CT-CT co-registration to determine technical success, we were able to identify a possible trend in the differentiation between patients with a very high risk (88.9%) of tumor recurrence and patients with low risk of recurrence (11.1%).

The superiority of quantitative assessment of minimal margins over qualitative visual assessment has also been demonstrated in patients with HCC. Kim et al. compared retrospectively qualitative visual assessment with quantitative, CT-CT fusion imaging, assessment in 103 patients undergoing RFA for 110 HCC lesions [7]. All 110 tumors were ablated with the intention to achieve a margin of > 5 mm. Yet, quantitative assessment using CT-CT fusion showed an ablation margin of > 5 mm in only 2.7% of the tumors, whereas based on visual assessment the percentage of ablations with > 5 mm margins was deemed to be 34.5%. The inaccuracy of visual assessment has also been demonstrated in a study by Park et al., in which qualitative assessment was compared with quantitative assessment in HCC patients by fusion of pre-ablation magnetic resonance imaging (MRI) and a post-ablation CT scan [8]. Quantitative assessments proved to be more accurately than qualitative assessment, especially when assessment was performed by radiologists with limited experience.

Against our expectations, in the patients in whom complete tumor ablation was achieved, the margin was below 5 mm (mean 2.2 ± 1.9 mm). These findings are in concordance with the previously mentioned study of Kim et al.. They found that a margin of ≥2 mm was sufficient to achieve LR of < 10% in patients with hepatocellular carcinomas. Although we cannot draw conclusions from our results due to the small patient number, this remarkable finding is in concordance with the data about LR and overall survival for surgical R0 resections of CRLM, for which a margin of 1 mm is required [12]. Another explanation might be that we did not take possible tumor shrinkage into account during measurements of the ablation and tumor borders. Therefore, obtained margins may have been wider in reality than how they were assessed in this study on the direct post-procedural CE-CT.

In future studies, quantitative analysis may be used to determine the endpoint of ablation and instigate immediate re-ablation when insufficient margins are obtained (while the patient is still under general anesthesia or sedation). In prospective studies mismatches between co-registration between pre- and post-ablation scans can be minimized by performing both scans immediately before and after ablation to ensure minimal difference in liver position and morphology. Minimizing breathing influences can be limited by using high-frequency jet ventilation or apnea during scanning [13]. Another feature that might increase the reliability of the Mirada RTx could be automatic evaluation of the delineated tumor and ablation zone. Since complete tumor ablation was in this study assessed by checking the projection of both borders in all scans visually.

## Conclusions

In conclusion, we showed that co-registration of pre- and post-ablation CT scans using Mirada RX is frequently not feasible as a result of differences in shape and position of the liver. Yet, in patients in whom accurate co-registration was feasible determination of technical success of ablation is reproducible and ablation margins correlated with LR. Future prospective studies should focus on optimized scanning protocols to increase feasibility of co-registration of pre- and post-ablation scans as quantitative assessment of ablation may prove to be a valuable tool in determining technical success and predicting LR.

## Additional file

Additional file 1: **Table S1.** Patient characteristics of all patients with percutaneous radiofrequency ablation for a solitary colorectal liver metastasis. **Table S2.** CT scanning protocol. (DOCX 16 kb)

## Data Availability

The datasets used and/or analysed during the current study are available from the corresponding author on reasonable request.

## Abbrevations

CEA: Carcinoembryonic antigen
CRLM: Colorectal liver metastasis
CT: Computer tomography
HCC: Hepatocellular carcinoma
LR: Local recurrence
MR: Multi resonance
PET: Positron emission tomography
RFA: Radio frequency ablation
SPECT: Single Photon Computed Tomography
