# Supplementary material for: Quantitative margin assessment of radiofrequency ablation of a solitary colorectal hepatic metastasis using MIRADA RTx on CT scans: a feasibility study
Source: BMC Med Imaging. 2019 Aug 20;19:71. doi: 10.1186/s12880-019-0360-2 (PMC6700773; doi:10.1186/s12880-019-0360-2)
Supplement: Supplementary file 1 — Table S1. Patient characteristics of all patients with percutaneous radiofrequency ablation for a solitary colorectal liver metastasis. Table S2. CT scanning protocol. (DOCX 16 kb) [file 12880_2019_360_MOESM1_ESM.docx]

SUPPLEMENTARY DATA

**Supplementary Table S1. Patient characteristics of all patients with percutaneous radiofrequency ablation for a solitary colorectal liver metastasis.**

|  |  |  | |
| --- | --- | --- | --- |
|  | | *n* |  |
| **total** |  | 29 |  |
| **age** | mean (SD) | 63.9 | ±11.4 |
| **sex** | male | 19 | 66% |
|  | female | 10 | 34% |
| **previous CRLM surgery** | yes | 11 | 38% |
|  | no | 18 | 62% |
| **occurrence of CRLM** | metachronous | 13 | 45% |
|  | synchronous | 16 | 55% |
| **year of RFA** | 2009-2011 | 12 | 41% |
|  | 2012-2014 | 17 | 59% |
| **lesion size (mm)** | median (range) | 21 | 8-42 |
| **follow-up (months)** | mean (SD) | 38.4 | ±19.5 |
| **survival** | death | 5 | 27% |

CRLM: colorectal liver metastasis

RFA: radiofrequency ablation

**Supplementary Table S2. CT scanning protocol**

Technique of Acquisition and Reconstruction:

| **Serie** | **kVp** | **Sure Expo Protocol** | **Delay pi (s)** | **Rot (s)** | **HP** | **Sure IQ – Reconstruction** |
| --- | --- | --- | --- | --- | --- | --- |
| Scano | 120 | 50/100 | - | - | - | Scano LD |
| Blanco | 120 | Q1 | - | 0.5 | 65 | Abdo / Stand |
| Testing | 100 | 50 | - | 1.0 | - | - |
| Perfusion | 100 | 100 | - | 0.5 | - | Body Perfusion |
| S&V | 120 | 40 | - | 0.5 | - | Abdo / Smooth |
| Sure Start | 120 | 80 | - | 0.5 | - | Abdo / Smooth |
| Late arterial | 120 | Q1 | SS +15 | 0.5 | 65 | Abdo / Stand |
| Portal venous | 120 | Q1 | SS + 50 | 0.5 | 65 | Abdo / Stand |
| Delayed | 120 | Q1 | 200 | 0.5 | 65 | Abdo / Stand |

Scan position:

| **Serie** | **Starting position** | **Final position** |
| --- | --- | --- |
| Scano | Symphysis pubis | Highest diaphragm |
| Blanco | Highest diaphragm | Lower edge liver |
| Testing | Mid liver | - |
| Perfusion | Tumor | - |
| S&V | Highest diaphragm | - |
| Sure Start | Aorta abdominalis | - |
| Late arterial | Highest diaphragm | Lower edge liver |
| Portal venous | Highest diaphragm | Lower edge symphysis pubis |
| Delayed | Highest diaphragm | Lower edge liver |

Archiving:

- Raw data to tapestreamer
- Dual scan
- Axial series 5mm/2.5mm
- Axial series 1mm/1mm
